# A Case of Anatopic Phenomenon in Dapsone Hypersensitivity Syndrome

**DOI:** 10.7759/cureus.67732

**Published:** 2024-08-25

**Authors:** Kartika Devi S., Divya Raviprakash, Anuradha Priyadarshini, Murugan Sundaram, Adikrishnan S.

**Affiliations:** 1 Dermatology, Sri Ramachandra Institute of Higher Education and Research, Chennai, IND

**Keywords:** anatopic response, lepromatous leprosy, dapsone hypersensitivity syndrome, sparing phenomena, anatopic phenomenon

## Abstract

We report a case of a 67-year-old woman on multidrug therapy for borderline lepromatous leprosy who developed dapsone hypersensitivity syndrome (DHS). She presented with fever and red pruritic rash over her face, neck, trunk and upper limbs, with distinct sparing of hypopigmented leprosy patches on her trunk. Laboratory findings showed anemia, elevated white blood cell count and liver function abnormalities. Upon discontinuation of dapsone and initiation of intravenous dexamethasone, the reaction subsided. This case report describes anatopic response, a phenomenon where a cutaneous infection modifies the inflammatory response of a secondary inflammatory dermatoses at the same location. We further elucidate the pathogenic mechanisms implicated in anatopic response, a phenomenon not commonly documented in the literature.

## Introduction

Anatopic phenomenon refers to the modification of the inflammatory response of one skin condition by an unrelated cutaneous illness at the same location while leaving the areas affected by the first disorder unaffected. This interesting phenomenon has been reported only rarely in literature involving infective primary dermatoses and non-infective secondary rash. Various theories have been proposed to explain the mechanism of anatopic phenomenon [1]. In this case report of dapsone hypersensitivity, we discuss a rare presentation of anatopic response in an elderly woman with borderline lepromatous leprosy.

## Case presentation

**Figure 1:**
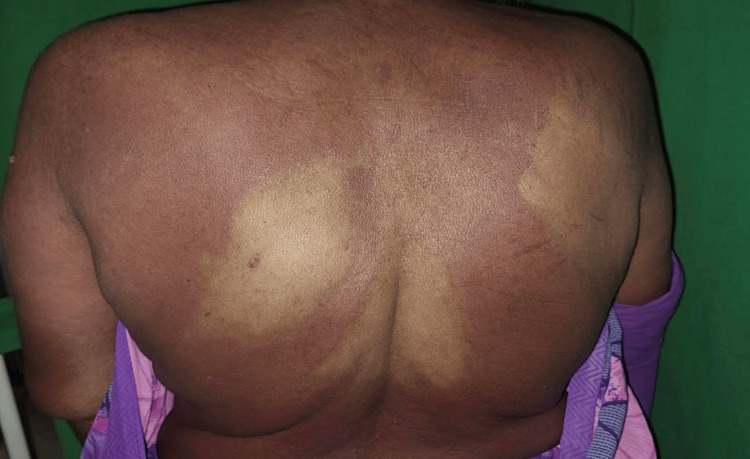
Clinical photos of the patient showing Hansen’s patches spared in DHS DHS: Dapsone hypersensitivity syndrome

**Figure 2:**
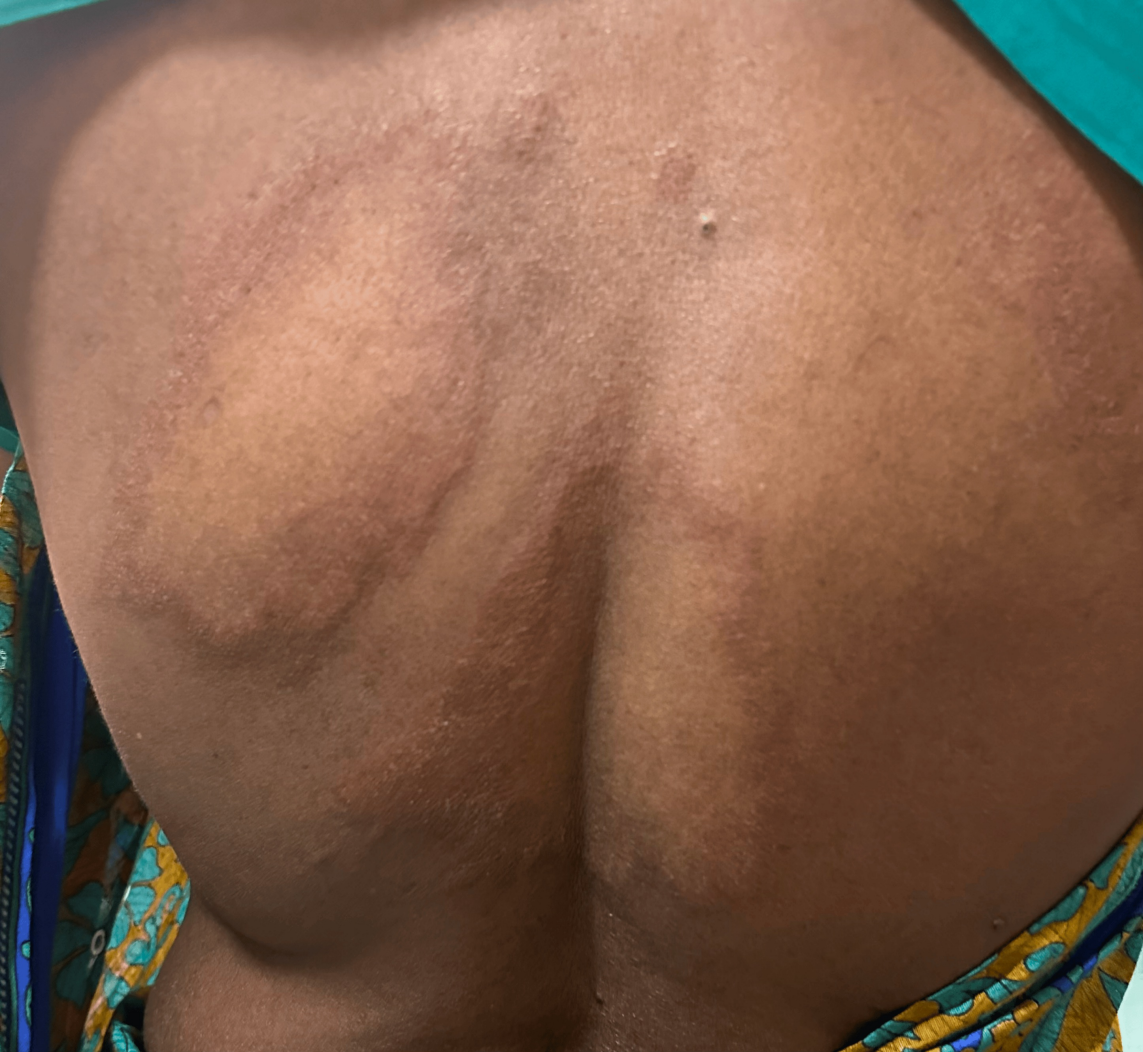
Clinical photos of the patient showing Hansen's patches four weeks after resolution of rash

A 67-year-old female who had been on multidrug therapy (rifampicin 600 mg and clofazimine 300 mg, supervised once a month; dapsone 100 mg and clofazimine 50 mg daily, unsupervised) for borderline lepromatous leprosy for one month presented with a history of a red pruritic rash and fever for five days. There were erythematous confluent macules and papules over the face, neck, trunk and both upper limbs, with sparse hypopigmented patches of Hansen’s disease on the trunk (Figures 1, 2). Xerosis and pitting pedal edema were noted over both lower limbs. Sensation was reduced over the dorsum of both feet; however, sensation over the lesions was preserved. Bilateral ulnar nerves were thickened. No motor changes were found in the clinical evaluation. Palms and soles were normal. Oral cavity examination revealed no erosions. The liver and spleen were not palpable. The cardiac and respiratory systems were unremarkable on examination. There was no palpable lymphadenopathy.

Investigation revealed reduced haemoglobin at 8.7 g/dL, elevated total WBC count at 14,900 cells/mm^3^ and an increased erythrocyte sedimentation rate of 33 mm/hour. Serum bilirubin was 1.26 mg/dL, and alanine transaminase was 103 units. The levels for urea, creatinine, uric acid and electrolytes were within normal limits. The ultrasound abdomen showed no abnormalities. A biopsy specimen from the hypopigmented patch taken earlier revealed normal epidermis, a clear grenz zone, macrophage granulomas with occasional epithelioid cells, and a few lymphocytes in the dermis around appendages. Acid-fast bacilli were abundant. A diagnosis of dapsone hypersensitivity syndrome (DHS) was considered based on the patient’s significant treatment history, clinical findings, deranged laboratory investigations and adherence to the Richardus and Smith criteria [2].

Dapsone was immediately stopped, and the patient was started on intravenous dexamethasone, ursodeoxycholic acid 300 mg and N. acetyl cysteine 600 mg twice a day. The rash and fever subsided over the next few days. She continued taking treatment consisting of anti-leprosy drugs without dapsone.

## Discussion

DHS, also known as fifth-week dermatitis, typically presents during the course of multibacillary multidrug therapy as an idiosyncratic reaction to dapsone. It is diagnosed based on Richardus and Smith criteria, which include the following: 1) symptoms appear within eight weeks after commencement of dapsone and disappear after discontinuation of the drug; 2) symptoms cannot be attributed to any other drug given simultaneously with dapsone; 3) symptoms are not attributable to lepra reaction; 4) no other disease liable to cause similar symptoms was diagnosed. A reaction is classified as DHS when symptoms start between the second and eighth weeks of treatment and at least two of the following signs or symptoms are present: fever, skin eruption, lymphadenopathy, liver pathology (hepatomegaly, jaundice and/or abnormal liver function tests) [2]. The pathogenesis behind DHS is not fully understood. Type 4 hypersensitivity has mainly been implicated, followed by type 1 and type 3 hypersensitivity. In our case, the rash presented as confluent erythematous macules and papules over the face, neck, trunk and upper limb, sparing the Hansen’s patches. 

This unique phenomenon of anatopic response occurs due to modulation of the inflammatory response of one dermatosis by another unrelated cutaneous infection at the same site. To our knowledge, the anatopic phenomenon has been reported in four other case reports of dapsone hypersensitivity in leprosy patients [3-6]. An extensive literature search revealed many other infective and non-infective dermatological conditions resulting in anatopic responses (Table 1). The first case of the sparing phenomenon was reported in the literature in 1987 in a tuberculoid leprosy patient with sparing of lesions due to ampicillin hypersensitivity [7]. Hansen’s disease and tinea versicolor are the primary dermatoses in which anatopic phenomenon have been described. The most common reason for rashes/eruptions is drug hypersensitivity, most commonly to dapsone (drug rash with eosinophilia and systemic symptoms (DRESS) syndrome), ampicillin and dianthrol, resulting in anatopic responses [3-5,7-8]. There have also been reports of conditions like acute generalised exanthematous pustulosis, polymorphous light eruptions, irritating contact dermatitis and viral exanthem with an anatopic response, with tinea versicolor being the primary dermatosis [1].

**Table 1:** List of various case studies in the literature reporting the occurrence of anatopic phenomena DHS: Dapsone hypersensitivity syndrome

Author & year of publication	Summary of case report	Primary dermatoses	Cause of rash formation	Geographic location of the reported case
Pakran and Riyaz [1] (2011)	A 25-year-old man diagnosed with acute generalized exanthematous pustulosis showed sparing of scaly lesions of tinea versicolor. A 28-year-old woman was diagnosed with polymorphous light eruptions with coexistent tinea versicolor. Hypopigmented scaly macules showed no signs of inflammation and were sharply demarcated. A 55-year-old man with acute irritant contact dermatitis presented with sparing of tinea versicolor lesions. An eight-year-old girl presented with discrete round and oval areas of sparing over the chest when diagnosed with viral exanthem with coexistent tinea versicolor.	Tinea versicolor	Acute generalized exanthematous pustulosis; Polymorphous light eruptions; Irritant contact dermatitis; Viral exanthem	India
Ng and Goh [3] (1998)	A 25-year-old male with hypoesthetic tuberculoid leprosy lesions developed generalized eruptions seven weeks post dapsone treatment with conspicuous sparing of leprosy lesions.	Tuberculoid leprosy	DHS	Singapore
Maddala et al. [4] (2017)	A 26-year-old male with borderline lepromatous leprosy presented with a one-week history of high fever, jaundice and a reddish itchy rash. Interestingly, scaly macules of tinea versicolor on the trunk were unaffected.	Borderline lepromatous leprosy & tinea versicolor	DHS	India
Gopinath et al. [5] (2017)	A 17-year-old male was diagnosed with borderline tuberculoid leprosy. The hypopigmented hypoanesthetic macules were spared in DHS.	Borderline tuberculoid leprosy	DHS	India
Hegde et al. [6] (2023)	A 50-year-old man with borderline leprosy presented with DHS with sparing of hypopigmented patches over right flank, anterior trunk and arm.	Borderline lepromatous leprosy	DHS	India
Pavithran and Bindu [7] (1987)	A middle-aged woman with hypopigmented patch of leprosy on the left cheek had sparing despite a generalized hypersensitivity eruption due to ampicillin.	Tuberculoid leprosy	Ampicillin hypersensitivity	India
Shenoy, Wali and Srinivas [8] (1993)	A 32-year-old male with dithranol-induced erythema had sparing of hypopigmented patches of tinea versicolor.	Tinea Versicolor	Dithranol-induced erythema	India
Sulaiman et al. [9] (2023)	A 20-year-old male diagnosed with pityriasis rosea showed selective sparing of the patches of pre-existing tinea versicolor.	Tinea versicolor	Pityriasis rosea	India
Nair and Kabilan [10] (2024)	A 62-year-old male with grouped vesicles of herpes zoster spared the areas affected by pityriasis versicolor in the neck and chest region.	Tinea versicolor	Herpes zoster	India

The expression of erythema as a part of cutaneous adverse drug reactions is a complex process resulting in dilation of the blood vessels supplying the skin. Cutaneous vasodilation is mediated by the release of several inflammatory peptides and neurogenic pathways [4]. Flare response refers to arteriolar and capillary vasodilation mediated by the axonal reflex through the release of vasodilator substances such as substance P, neurotensin and kinin. Thus, it represents neurogenic vasodilatation and is considered to be a function of an intact nervous system [11].

Several patho-mechanisms have been discussed to explain anatopic phenomena. The most widely accepted theory is that nerve damage caused by leprosy affects neuronally mediated cutaneous vasodilation, resulting in the impaired ability of the skin to flare up in response to histamine. Nerve damage also results in a downregulation of mast cell activity. These mast cells are responsible for the release of various vasoactive amines that act on the cutaneous microvasculature. So, a lack of these vasodilatory peptides contributes to the absence of cutaneous reactions at the sites of anatopic phenomena. Furthermore, it is proposed that substance P also plays a major role in cellular proliferation and immunostimulation. It also improves monocyte chemotaxis, cytokine release and production, and T-cell proliferation. Hence, leprosy-related nerve injury impacts not only the microvasculature but also diminishes the local immune response, leading to the sparing of leprosy patches in this hypersensitivity reaction [3,4]. The concept of immunocompromised cutaneous district is used to denote a particular area of immunologic dysregulation in an otherwise immunocompetent individual. This type of regional immune dysfunction is caused by a sectoral failure of lymph flow that affects the influx of immune cells and/or damage to cutaneous nerves that results in altered neuropeptide release [12].

**Figure 3:**
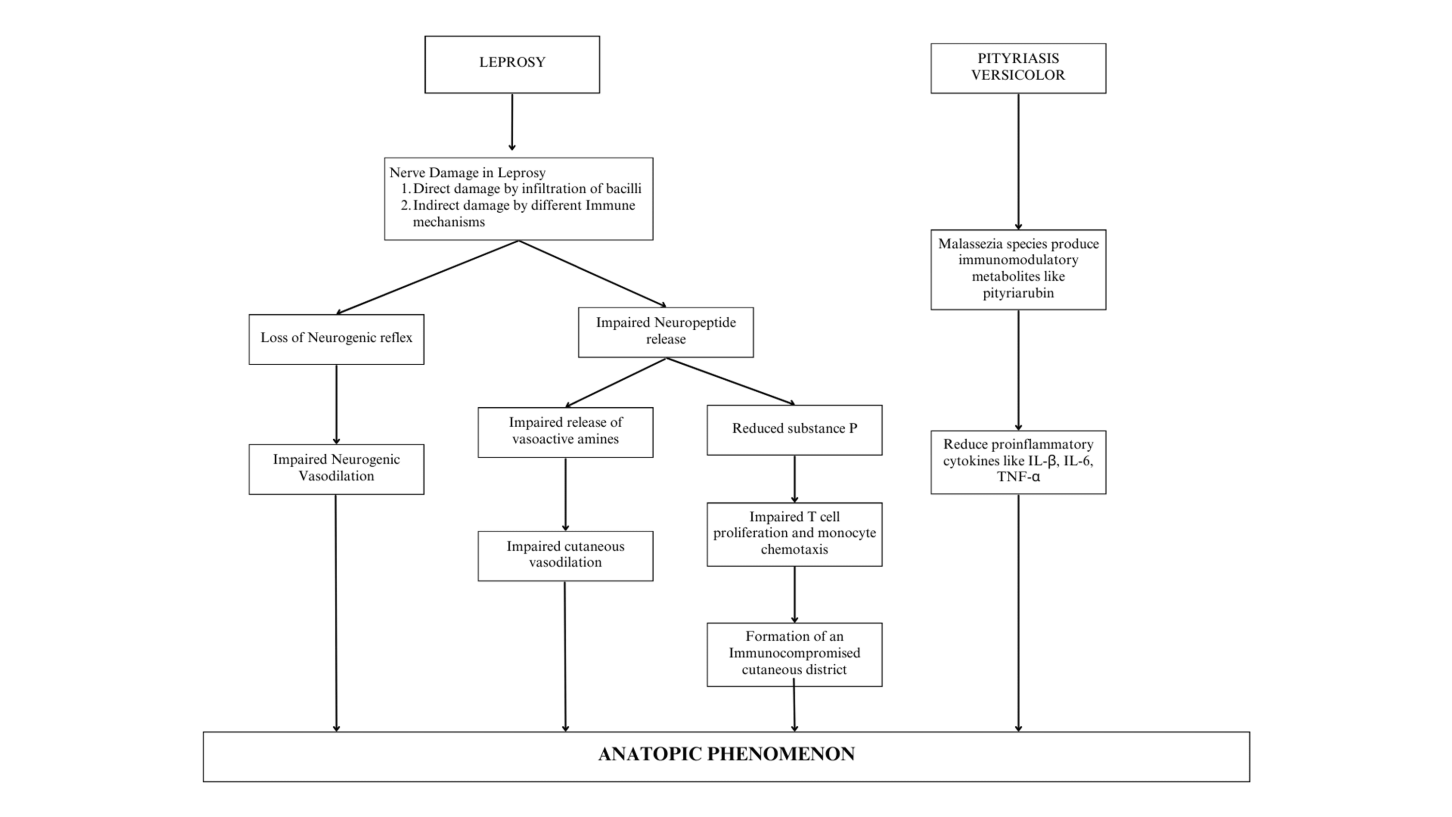
Summary of the patho-mechanism of the anatopic phenomenon

Anatopic phenomena have also been described in tinea versicolor lesions. Malassezia species produces several metabolites, such as pityriarubin, which acts by modulating pro-inflammatory and immunomodulatory cytokines. In vitro, Malassezia reduces local pro-inflammatory cytokines such as IL-1beta, IL-6 and TNF-alpha, resulting in anatopic phenomena (Figure 3) [1,13].

## Conclusions

The anatopic phenomenon refers to the localized alteration of the inflammatory response in one skin disorder due to the presence of another unrelated cutaneous condition at the same site. This case report highlights the rare occurrence of this phenomenon in a patient with DHS in a patient of borderline lepromatous leprosy. The selective sparing of leprosy patches in the presence of a generalized drug-induced rash highlights the complex interplay between local immune responses and neurogenic factors, potentially driven by leprosy-related nerve damage and the down-regulation of mast cell activity. The molecular mechanisms of anatopic response need to be explored further.
